# Strategy for the creation of clinical grade hESC line banks that HLA-match a target population

**DOI:** 10.1002/emmm.201201973

**Published:** 2012-11-19

**Authors:** Laureen Jacquet, Emma Stephenson, Robert Collins, Heema Patel, Jane Trussler, Roaa Al-Bedaery, Pamela Renwick, Caroline Ogilvie, Robert Vaughan, Dusko Ilic

**Affiliations:** 1 Embryonic Stem Cell Laboratories, Guy's Assisted Conception Unit, Division of Women's Health, King's College School of MedicineLondon, UK; 2 Clinical Transplantation Laboratory, GSTS Pathology, Guy's HospitalLondon, UK; 3 Guy's and St. Thomas' Centre for Preimplantation Genetic Diagnosis and Genetics Centre, Guy's and St. Thomas' NHS Foundation TrustLondon, UK; 4 MRC Centre for Transplantation, King's College London, King's Heath PartnersLondon, UK

**Keywords:** clinical grade stem cells, clinical grade human embryonic stem cell validation and banking, HLA typing, human embryonic stem cells, single cell analyses

## Abstract

Here, we describe a pre-derivation embryo haplotyping strategy that we developed in order to maximize the efficiency and minimize the costs of establishing banks of clinical grade hESC lines in which human leukocyte antigen (HLA) haplotypes match a significant proportion of the population. Using whole genome amplification followed by medium resolution HLA typing using PCR amplification with sequence-specific primers (PCR-SSP), we have typed the parents, embryos and hESC lines from three families as well as our eight clinical grade hESC lines and shown that this technical approach is rapid, reliable and accurate. By employing this pre-derivation strategy where, based on HLA match, embryos are selected for a GMP route on day 3–4 of development, we would have drastically reduced our cGMP laboratory running costs.

## INTRODUCTION

Cell therapy has to compete with other technologies in a range of criteria including cost. Encouraging initial clinical trials have brought the reality of human embryonic stem cell (hESC)-based therapy a significant step closer (Brindley & Mason, 2012; Ilic & Polak, 2012; Schwartz et al, 2012). However, for hESC to be used in a wide range of treatments, it is imperative to minimize immune responses following transplantation of differentiated progeny. One strategy is to establish clinical-grade hESC banks with human leukocyte antigen (HLA) haplotypes, which will match a significant proportion of the population. The limitations are the substantial investments of time and money required to derive hESC lines under current Good Manufacturing Practice (cGMP)-compliant conditions. Current techniques of embryo HLA typing are lengthy and require validation of each family at the single cell level. Legislation protecting anonymousness of embryo donors for stem cell research makes this approach unworkable. We have developed an embryo pre-selection method that provides medium resolution HLA results from a single cell in as little as 9 h post-biopsy, with no need for parental tissue samples, maximizing the efficiency and minimizing the costs of establishing such banks. We have validated our strategy by analysing parents, embryos and hESC lines from three families demonstrating that this technical approach is rapid, reliable and accurate.

Cells targeted by the immune system usually express HLA or minor histocompatibility (H) antigens. These, along with ABO blood group antigens are crucial factors in the immune response to transplantation. Undifferentiated hESC express low level HLA class I but no HLA class II molecules, with some also expressing H antigens (Drukker et al, 2002; Taylor et al, 2011). Upon differentiation, both *in vitro* and *in vivo*, the expression of HLA class I molecules is upregulated as it also occurs along normal developmental pathways (Drukker et al, 2002). This therefore renders the cells susceptible to immunological rejection.

Several strategies for preventing immune recognition of hESC have been suggested. Inducing peripheral tolerance in the intended recipient by haematopoietic chimerism has received much attention (Batten et al, 2007; Chidgey et al, 2008; Lui et al, 2009; Sordi and Piemonti, 2011). Other approaches include the generation of isogenic hESC lines by somatic cell nuclear transfer, use of homozygous parthenogenetic hESC lines, transplantation of hESC into immunoprivileged sites, genetic modification of hESC to reduce the expression of HLA molecules or the long-term use of immunosuppression (Batten et al, 2007; Lui et al, 2009; Revazova et al, 2008).

Establishing banks of immunophenotyped lines offers a straightforward approach to addressing the issue of cell rejection and is based on decades of experience with cell and organ donation. The major drawback is the phenomenal investment necessary to establish and maintain cGMP facilities and the intense workload involved in the derivation, propagation and characterization of numerous lines in the hope of obtaining those with a common HLA combination. In an estimate of the number of lines required in the UK, it has been calculated that 150 lines from random donors would match 38% of the population with a single HLA mismatch or better (Taylor et al, 2005). In a similar exercise for Japan, a theoretical bank containing 170 hESC lines from randomly selected donated embryos was estimated to provide a single mismatch or better for 80% of the population (Nakajima et al, 2007). The relatively low ethnic diversity in the Japanese population contributes to this high matching potential. In Korea, 28 existing hESC lines were evaluated for potential suitability for transplantation. When two HLA mismatches were allowed at the A, B, DRB1 allele level, it was estimated that 16% of the possible recipients could be matched to one or more donor cell lines (Lee et al, 2010). The first Brazilian hESC line, BR-1, and 22 hESC lines established elsewhere, matched only 0.011% of the Brazilian population (Fraga et al, 2011). Hence, the number of lines required when derivation is random with respect to HLA is likely to be prohibitive when the costs necessary to derive them under cGMP-compliant conditions are calculated. Worldwide, there are several million potential beneficiaries of stem cell therapy, including patients suffering from neurodegenerative, autoimmune, cardiovascular and haematopoietic diseases (Taylor et al, 2011). In order to realize the full potential of this therapeutic approach, treatment must be affordable on a large scale.

To assess the potential suitability of hESC lines, HLA typing for HLA-A, -B, -C, and DRB1 can be performed and the results entered into the MatchView® database (Be the Match Registry, http://marrow.org/Patient/Transplant_Process/Search_Process/View_Potential_Matches/View_Potential_Matches.aspx, Accessed 17 May 2012). MatchView® shows potential matches on the Be The Match® Registry at the moment the tool is used and is updated constantly. The registry includes 9.5 million adult donors (NMDP Registry for Potential Adult Donors) and more than 165,000 searchable cord blood units (NMDP Registry for Potential Cord Blood Units). Generally, 6 of 8 markers are required for matching adult donors at HLA-A, -B, -C and -DRB1 and 4 of 6 for matching cord donors at HLA-A, -B and -DRB1. The HLA system is the most polymorphic of all human genetic systems and this diversity raises a substantial challenge in the identification of matched donors. At 12 HLA loci 5880 different alleles at HLA class I and 1647 at HLA class II have been identified and the list continues to grow (International Immunogenetics, http://www.ebi.ac.uk/imgt/hla/stats.html, Accessed 23 May 2012). The most important HLA molecules to match are the class I HLA-A and HLA-B and the class II HLA-DR. A total of 1365 different HLA-A proteins, 1898 different HLA-B and 821 different HLA-DR are currently identified (International Immunogenetics). Although it may be rare that a cell bank will provide a perfect match, it is reasonable to expect that partial matching will help survival of the transplanted cells and could reduce the need for immunosuppressive therapy (Taylor et al, 2005). Certainly in allograft organ transplantation, there is an increase in graft survival as the degree of HLA disparity is reduced (Opelz et al, 1999). The overall consensus for the use of hESC in most circumstances is that the HLA matching can be made at a similar level to solid organ transplantation with the addition of immunosuppression (Taylor et al, 2011). Only the recipient response to the stem cells is of initial importance as hESC are not directly immune active. Accordingly HLA typing at medium resolution was used to classify the hESC lines in the Be The Match® Registry. hESC with a match level of 1/10,000 or more at the HLA-A, -B and -DRB1 loci were considered of prime importance. Match levels between 1/10,000 and 1/100,000 might be considered for further banking if funding is available. After selection and expansion, DNA from the chosen hESC can be HLA typed at high resolution so that they can be made available for haematopoietic purposes.

## RESULTS AND DISCUSSION

**Figure 1:**
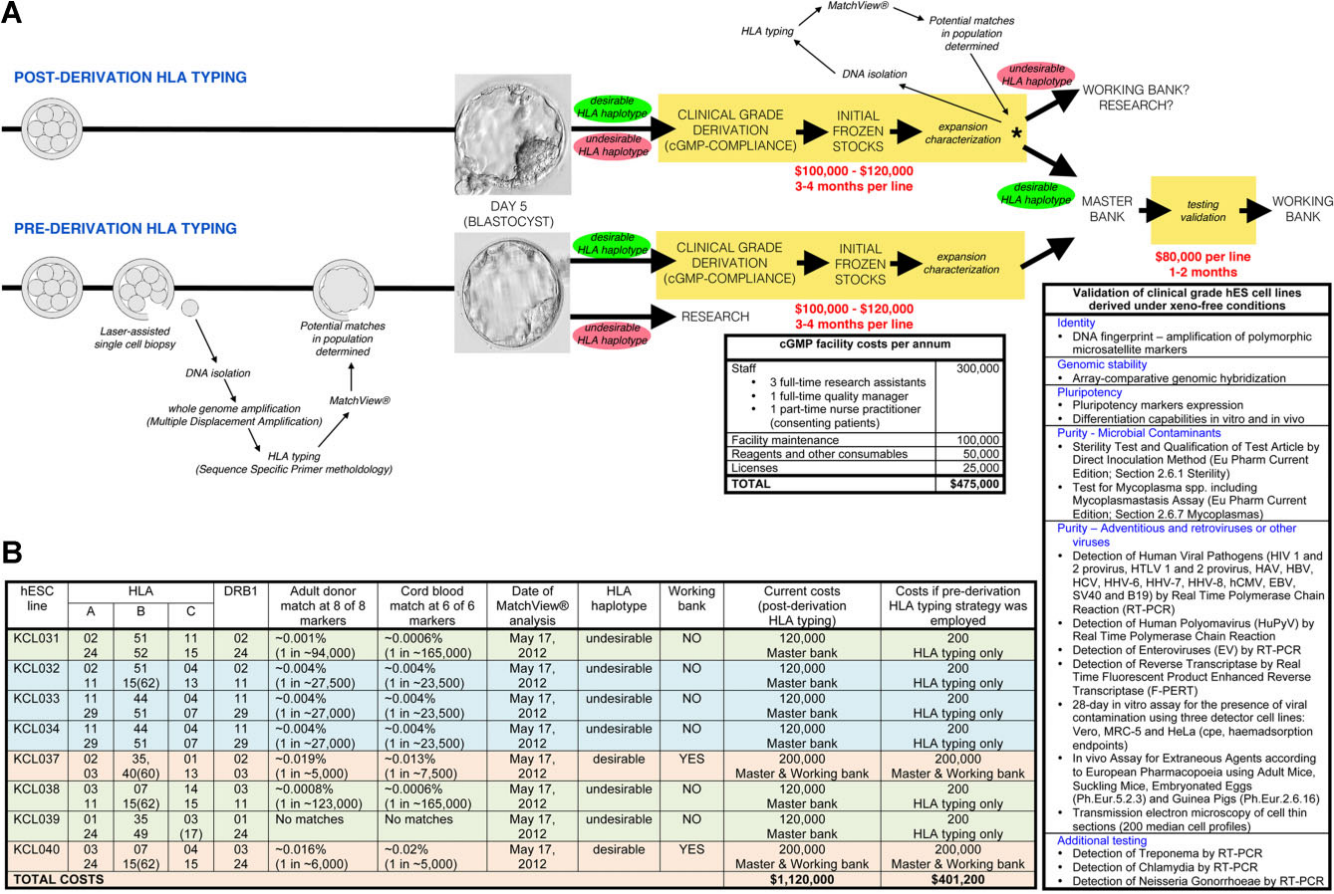
Post- *versus* pre-derivation haplotyping strategy for the generation of clinical grade hESC lines Using post-derivation haplotyping, every line is derived and cultured in cGMP conditions at the cost of around 120,000 USD per line. From analysis of initial frozen stocks, only those *lines* with desirable haplotypes are taken forward for further validation and banking. Using pre-derivation haplotyping, only those embryos with desirable haplotypes are used for derivation in cGMP conditions. This approach offers significant cost savings as well as a more efficient use of time, labour and resources. A breakdown of the annual cGMP laboratory running costs and the validation tests performed on the Master Bank are shown. Cell line identity, genomic stability and pluripotency are also assessed at the initial frozen stock stage, as shown in Supporting Information Figs 1 and 2.Desirability of our clinical grade lines. The percentage of the population that could be fully matched at the common HLA loci was determined for our clinical grade cell lines. KCL037 and KCL040 are common, matching significantly more than 1/10,000 people and are therefore considered desirable. All the other lines are considered undesirable. With the pre-derivation strategy, we would have used only those embryos that gave rise to KCL037 and KCL040 in the cGMP route, saving our laboratory in the region of 700,000 USD.

In our cGMP-compliant licensed facility, we have recently derived eight clinical grade hESC lines (Ilic et al, 2012; Stephenson et al, 2012; Supporting Information Fig 1). HLA typing cells from our initial frozen stocks provided an interesting set of data (Fig 1B; Supporting Information Table 1). Based on results from the NMDP database, our therapeutic bank has two extremes: at best, KCL037 and KCL040 are relatively frequent and would fully match 1 in 5000–7000 of the population and, at worst, KCL039 is very rare and would match none. From one standpoint, it could be argued that because of its infrequent combination of HLA haplotypes KCL039 is exceptionally valuable. Indeed, strategies are in place in bone marrow transplantation centres to selectively recruit stem cell donors with rare HLA phenotypes (Schmidt et al, 2007). From another, more practical point of view, it is highly undesirable to have such rare combination in a bank of limited size that aims to match the largest proportion of the population as possible. However, the least desirable result is that two of our lines, KCL033 and KCL034, have identical HLA haplotypes. This is not overly surprising. The lines are derived from embryos from the same donor couple and therefore the probability that HLA haplotypes will be identical is 1 in 4. Since couples undergoing IVF commonly end up with more than one supernumerary embryo, sibling lines are frequently derived when these embryos are donated for stem cell research.

The frequency of HLA haplotypes will influence our decision as to which of these lines will be expanded from initial frozen stocks to create a Master Cell Bank and undergo additional validation that includes testing for contaminants, viruses and other adventitious agents. Testing itself takes around 1–2 months and runs at around 80,000 USD; preparation of the Working Cell Bank takes an additional 3–4 months. Obviously, such strategic planning based on the HLA results of newly derived cell lines could result in substantial savings. However, the expense is still exceedingly high. The costs of deriving and generating the initial frozen stock for one clinical grade hESC line under cGMP-compliant conditions, including maintenance of the facility and labour, are somewhere in range of 100,000–120,000 USD. Clearly, there is an urgent need to increase diversity, maximize efficiency, conserve resources and reduce capital investment in the establishment of clinical grade hESC line banks.

Assisted Conception Centers such as ours, that have an ethnically diverse population of patients, are ideally suited for consenting donors and procurement of supernumerary embryos for stem cell research (Supporting Information Table 2). We have addressed the need for HLA diversity and preserving costs by developing a new strategy of HLA haplotype-based pre-selection of those embryos that will be used for derivation of clinical grade lines in our cGMP-compliant facility. We use a preimplantation genetic diagnosis (PGD) approach to determine the HLA haplotypes of the embryos. PGD was developed as an alternative to prenatal screening for couples known to carry monogenic diseases or chromosomal translocations (Braude et al, 2002; Renwick et al, 2006). The method enables the selection of unaffected embryos for transfer, hence avoiding the risk of pregnancy termination and has become an integral part of assisted reproductive services. Recently, this technology has been applied to the testing of HLA type in order to select embryos that are tissue matched to an existing sibling in need of a stem cell transplant, so-called ‘saviour siblings’. This application has been performed in combination with PGD or without identification of a causative gene as a stand-alone test (Verlinsky et al, 2001, 2004). For either approach, the manipulation of the embryo is identical. On day three of development (eight-cell stage), one cell is removed from the embryo and taken for genetic analysis; the embryo is returned to culture and its development monitored. Those embryos that reach the blastocyst stage and are found to be of the required genetic status are transferred to the patient or cryopreserved for future use.

We have modified this strategy to develop pre-derivation HLA typing for all embryos donated for stem cell work. Those embryos that have a desirable HLA profile (a match of 1/10,000 or more) are used for clinical grade derivation attempts. As the medium resolution HLA results are available in as little as 9 h post-biopsy, the embryos biopsied at day 3 can be used for derivation attempts from day 5 onwards as routine. Those embryos that do not meet this requirement can then be used to derive research grade lines without the stringency and cost required for cGMP (Fig 1A).

**Figure 2:**
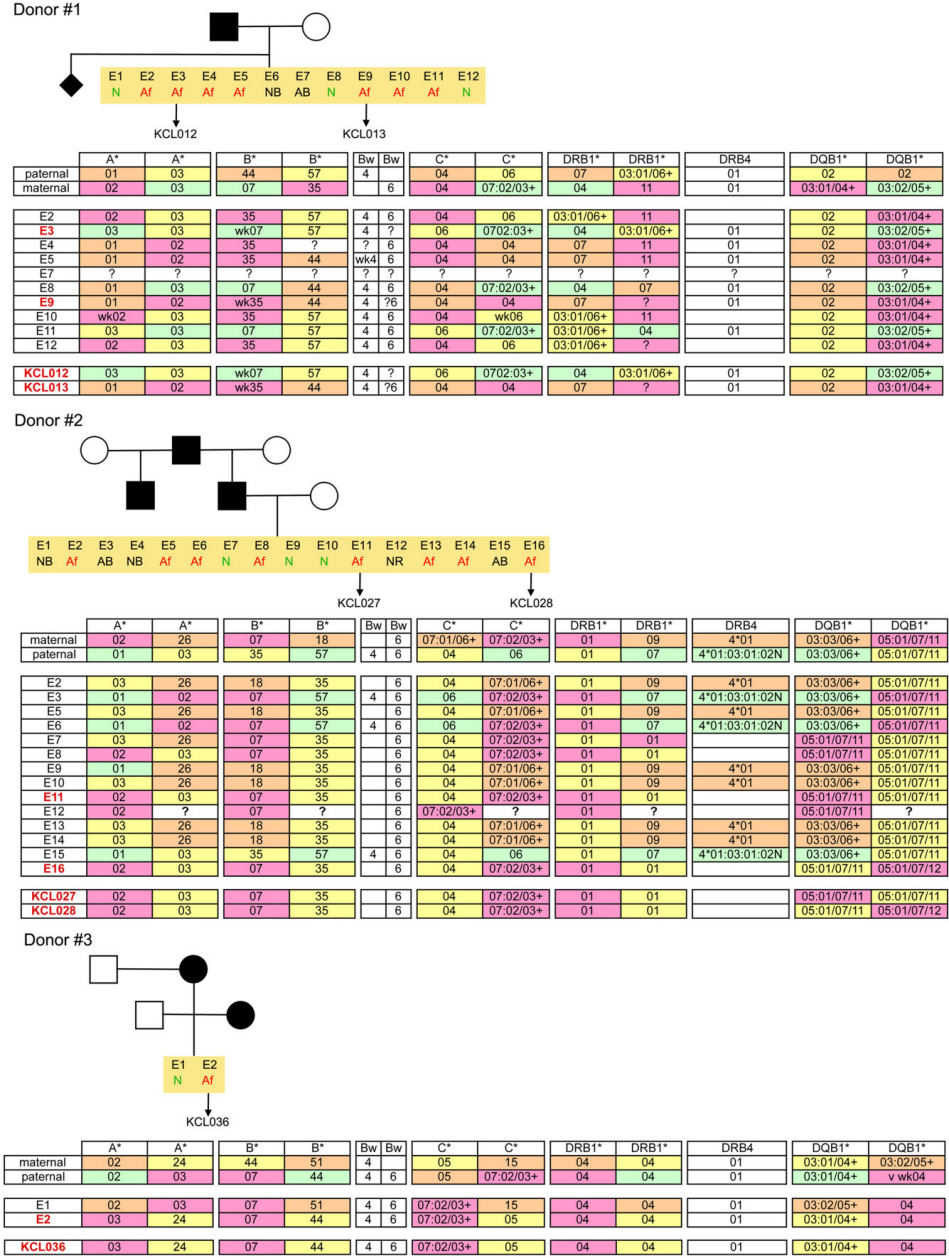
Genetic pedigree trees, embryo diagnosis and HLA haplotypes outcome for three couples undergoing PGD for HD Donor couple #1 had 12 embryos created, of which 11 were suitable for biopsy on day 3. Three were diagnosed as unaffected with HD, seven were diagnosed as affected and one had an abnormal result (maternal signal only). All seven affected embryos were donated to research; six progressed to the blastocyst stage and two hESC lines, KCL012 and KCL013, were derived. Donor couple #2 had 16 embryos created, of which 14 were suitable for biopsy on day 3. Three were diagnosed as unaffected with HD, eight were diagnosed as affected, two had an abnormal result at analysis (one with a maternal signal only, one with a paternal only) and one gave no result. The affected embryos were donated to research; three progressed to the blastocyst stage and two hESC lines, KCL027 and KCL028, were derived. Donor couple #3 had two embryos created, both suitable for biopsy on day 3. One was diagnosed as unaffected with HD, one as affected. The affected embryo was donated to research; it progressed to the blastocyst stage and the hESC line KCL036 was derived. Identical HLA haplotypes were obtained for each hESC line and its embryo of origin. KCL027 and KCL028 were derived from sibling embryos and share identical HLA haplotypes. AB, abnormal result; N, normal (embryo does not carry abnormal HD gene); Af, affected; NB, not biopsied; NR, no result.

We validated this approach with three couples that underwent PGD for Huntington's disease (HD) in our facility and who had hESC lines derived from affected embryos that were donated for stem cell research (Fig 2; Supporting Information Fig 2). Donor couple #1 had seven affected embryos from which two hESC lines, KCL012 and KCL013, were derived (Ilic et al, 2012). Donor couple #2 had eight affected embryos from which two lines, KCL027 and KCL028, were derived. Donor couple #3 had one affected embryo, from which KCL036 was derived. HLA typing was performed on blood samples from the couples, on the hESC lines and on DNA from the biopsied cells of the embryos. Identical HLA haplotypes were obtained for each embryo-of-origin and hESC line pair, showing that the technical approach to pre-derivation haplotyping is robust and accurate (Fig 2).

A single blastomere contains ∼6 pg of DNA, making detailed genomic analysis extremely challenging. Published reports of embryo selection for HLA status use nested and hemi-nested PCR for selected STR markers in the HLA region (Verlinsky et al, 2001, 2004); this approach requires lengthy genotyping, optimization and validation of each family at the single cell level. Our PGD program uses whole genome amplification prior to genetic testing, which typically gives microgram quantities of DNA, sufficient for single nucleotide polymorphism (SNP)-typing for HLA status as well as the haplotype status for any disease gene being tested.

Using results from 5 hESC lines, 27 embryos and 3 donor couples, we have shown here that this approach is reliable and accurate and can easily be performed in the time frame required to use fresh embryos for derivation. In our particular case, employing this strategy would have reduced our expenditure by around 75%, a saving of approximately 700,000 USD, as our aim is to derive lines with HLA haplotypes that are the most frequent in any ethnicity. We therefore would have taken only KCL037 and KCL040 through to clinical grade production (Fig 1b). With a small bank, we have chosen a matching level of 1/10,000 of the population to continue with clinical grade banking, with a larger bank this could be extended to 1/100,000 (which would include KCL032 and KCL033 or KCL034). To incorporate particularly rare phenotypes, perhaps 1 in 250,000 could be considered, but justifying the investment for this level of therapeutic potential may be difficult. Costing around 200 USD, our HLA haplotyping strategy has the potential to drastically reduce the capital investment required to populate clinical grade hESC banks with therapeutically relevant lines, by pre-selecting those embryos for derivation with desirable HLA phenotypes.

## MATERIALS AND METHODS

The work described here is done under licence from the UK Human Fertilisation and Embryology Authority (research licence numbers: R0075 and R0133) and also has local ethical approval (UK National Health Service Research Ethics Committee Reference: 06/Q0702/90). Informed consent was obtained from all subjects and the experiments conformed to the principles set out in the WMA Declaration of Helsinki and the NIH Belmont Report. No financial inducements are offered for donation.

### Derivation of hESC lines

The methods have been previously described in detail (Ilic et al, 2012; Stephenson et al, 2012).

### HLA typing of HD case

Preimplantation genetic diagnosis is used in couples who are at risk of transmitting a genetic disorder to their offspring. Various methods are used across PGD centres, often dictated by national regulations. At Guy's Hospital, we routinely biopsy a single blastomere from cleavage-stage embryos (day 3 post-fertilization). Once the DNA is isolated from the blastomere and subjected to whole genome amplification by multiple displacement amplification (MDA), haplotype analysis is performed in order to identify specific mutation-carrying embryos. hESC are then derived from embryos unsuitable for clinical use, carrying the familial mutation, and that are donated for research by consented couples.

**The paper explained**

PROBLEM:

Worldwide, there are several million potential beneficiaries of hESC therapy, including patients suffering from neurodegenerative, autoimmune, cardiovascular and haematopoietic diseases. In order to realize the full potential of this therapeutic approach, two major hurdles must be addressed: treatment needs to be affordable on a large scale, and immune responses following transplantation of differentiated progeny must be minimized. Whilst the approach to establish clinical grade hESC banks with HLA haplotypes which match a significant proportion of the population addresses the issue of cell rejection, it is limited by the substantial investments of time and money required to derive hESC lines under cGMP-compliant conditions and the intense workload involved in the derivation from embryos, then propagation and characterization of numerous lines in the hope of obtaining those with a common HLA combination. There is an urgent need to increase diversity, maximize efficiency, conserve resources and reduce capital investment in the establishment of clinical grade hESC line banks.

RESULTS:

We have developed an embryo pre-selection method that provides medium resolution SNP HLA results from a single cell (blastomere) in as little as 9 h post sample collection, with no need for parental tissue samples. Therefore, those embryos with a desirable HLA status (matching 1/10,000 of the population) can be identified extremely early and taken through to cGMP derivation of an hESC line, thus maximizing the efficiency and minimizing the costs of establishing therapeutically relevant banks. Those embryos with an undesirable HLA status can be used for other research. We have validated our strategy by analysing parents, embryos and hESC lines from three families demonstrating that this technical approach is rapid, reliable and accurate.

IMPACT:

The current approach to HLA typing from preimplantation embryos requires lengthy genotyping, optimization and validation of each family at the single cell level. We have demonstrated here for the first time that whole genome amplification of genomic DNA from a single blastomere is sufficient for rapid, reliable and accurate SNP typing for medium resolution typing by PCR amplification with sequence-specific primers. Employing this strategy has the potential to drastically reduce expenditure; at around 200 USD, HLA typing the embryo can avoid the cost of 100,000–120,000 USD needed to generate initial stocks of a line which does not match a significant proportion of the population. The capital investment required to populate clinical grade hESC banks with therapeutically relevant lines is therefore significantly reduced, by pre-selecting those embryos for derivation with desirable HLA phenotypes – hence addressing both the cost of treatment and the need to minimize immune rejection upon transplantation.

### Preimplantation genetic haplotyping for HD

Biopsied blastomeres were washed through polyvinylpyrrolidone/phosphate-buffered saline (PBS) and collected in 2.5 µl of 200 mM NaOH, 50 mM dithiothreitol using a fine polished glass capillary and overlaid with mineral oil. The cells were then lysed by incubation at 65°C for 10 min and the lysate was then neutralized with 2.5 µl of 200 mM tricine before undergoing MDA using the REPLI-g Midi kit (QIAGEN) by adding 45 µl of reaction master mix (15 µl nuclease-free water, 29 µl REPLI-g Midi reaction buffer, 1 µl REPLI-g Midi DNA polymerase), then incubated at 30°C for 4 h followed by inactivation at 65°C for 3 min and stored at 4°C until PCR analysis was performed.

Polymorphic markers flanking and intragenic to the Huntingtin (HTT) gene on chromosome 4p16.3, were identified using the UCSC Genome Browser (http://www.genome.ucsc.edu). Primers were then designed for 15 microsatellite markers using Primer3 software (http://frodo.wi.mit.edu/primer3) and checked for the presence of SNPs using the SNPCheck bioinformatics program from UK National Genetics Reference Laboratory (https://ngrl.manchester.ac.uk/SNPCheckV2.1/snpcheck.htm) and for human ALU repeats using a nucleotide BLAST search (http://blast.ncbi.nlm.nih.gov/Blast.cgi). The primers were designed to enable testing for inheritance of the at risk HD allele using two multiplexes, with each marker designed to amplify a different PCR product size and assigned a tag (6FAM™, VIC®, NED™ or PET™ dyes; Applied Biosystems) to ensure no overlap in marker fragments. A universal tagged primer approach was used (Heath et al, 2000; Pagan et al, 2004). PCRs were performed in 10 µl reactions containing DNA, 0.4 µl of an optimized non-fluorescent tagged primer mix, 4 pmol fluorescently labelled universal tag primer mix, and 5 µl of 2× QIAGEN Multiplex PCR Master Mix (containing HotStarTaq® DNA Polymerase, Multiplex PCR Buffer with 6 mM MgCl_2_, dNTP mix). Amplifications were performed on a G-Storm 2 thermal cycler using the following conditions: initial denaturation of 95°C for 15 min, followed by 29 cycles of 94°C for 30 s, 59°C for 60 s, 72°C for 60 s; then 72°C for 5 min, followed by 60°C for 20 min. PCR products were run on an ABI3730 genetic analyser using POP-6 polymer with a Genescan-600 LIZ size standard (Applied Biosystems) and analysed using GeneMarker (SoftGenetics), with the haplotypes being constructed manually from the genotype data.

### HLA typing

Pre-derivation HLA-class I typing for the embryos was carried out using PCR amplification with sequence-specific primer (PCR-SSP) based on the method of Bunce et al (1995). Ninety-six individual primer mixes were incubated with 10 ml of a mix containing approximately 10 ng test DNA; 1× PCR Buffer IV (Abgene, UK); 1.08 mM MgCl2 (Abgene, UK); 0.2 mM dNTP mix (Abgene, UK); 0.33 units of Taq DNA polymerase (Abgene, UK) and 57 nM stock of control primers (63/64) that recognize conserved homologous sequences in exon 3 of the HLA-DRB1 gene (Oswel, Southampton, UK). PCR amplifications were carried out in Applied Biosystems 2720 thermocyclers (Applied Biosystems, UK) using the following conditions: one cycle at 94.0°C for 60 s; five cycles at 94.0°C for 25 s, 70.0°C for 45 s and 72.0°C 30 s followed by twenty cycles at 94.0°C for 25 s, 63.0°C for 45 s and 72.0°C for 30 s and then five cycles at 94.0°C for 25 s, 55.0°C for 60 s and 72.0°C for 120 s. HLA class I allele and group-specific PCR amplicons were identified by gel electrophoresis in 1.0% agarose gels containing 1 µg/L ethidium bromide in TBE buffer. A digital image of each gel was obtained using UV illumination and a UVP Imagestore 7500. The relative size of the HLA class I specific amplicons was determined by comparison against the migration of a 100–1000 bp DNA molecular weight marker ladder (Abgene, UK). Successful PCR amplifications were verified with the 63/64 control primers, which generate a 784 bp amplicon.

Pre-derivation HLA-class II typing for the embryos was carried out using PCR-SSP based on the methods of Olerup & Zetterquist (1992); Olerup et al (1993) and Bunce et al (1993). Thirty-six individual primer mixes were incubated with 10 ml of a mix containing approximately 20 ng test DNA; 1× PCR Buffer IV (Abgene, UK); 1.08 mM MgCl2 (Abgene, UK); 0.2 mM dNTP mix (Abgene, UK); 0.33 units of Taq DNA polymerase (Abgene, UK) and 57 nM stock control primers that recognize conserved homologous sequences in the human growth hormone (HGH) gene (Oswel, Southampton, UK). PCR amplifications were carried out in Applied Biosystems 2720 (Applied Biosystems, UK) or MJ Research PTC 200 thermocyclers (GRI, UK) using the following conditions: one cycle at 94.0°C for 5 min and 30 cycles at 94.0°C for 25 s, 64.0°C for 50 s and 72.0°C 35 s. HLA class II allele and group-specific PCR amplicons were identified by gel electrophoresis in 1.0% agarose gels containing 1 µg/L ethidium bromide in TBE buffer. A digital image of each gel was obtained using UV illumination and a UVP Imagestore 7500. The relative size of the HLA class II specific amplicons was determined by comparison against the migration of a 100–1000 bp DNA molecular weight marker ladder (Abgene, UK). Successful PCR amplifications were verified with the HGH control primers, which generate a 485 bp amplicon.

## Supplementary material

Detailed facts of importance to specialist readers are published as ”Supporting Information”. Such documents are peer-reviewed, but not copy-edited or typeset. They are made available as submitted by the authors.
