# Supplementary material for: Strategy for the creation of clinical grade hESC line banks that HLA-match a target population
Source: EMBO Mol Med. 2012 Nov 19;5(1):10–7. doi: 10.1002/emmm.201201973 (PMC3569650; doi:10.1002/emmm.201201973)
Supplement: Supplementary file 1 [file emmm0005-0010-SD1.pdf]

EMBO Molecular Medicine

Manuscript EMM-2012-01973

Strategy for the creation of clinical grade hESC line banks that HLA-match a target population

Laureen Jacquet, Emma Stephenson, Robert Collins, Heema Patel, Jane Trussler, Roaa Al-Bedaery, Pamela Renwick, Caroline Ogilvie, Robert Vaughan and Dusko Ilic

Corresponding author: Dusko Ilic, King's College London

Review timeline:

Submission date:	03 September 2012
Editorial Decision:	01 October 2012
Revision received:	04 October 2012
Accepted:	09 October 2012

Transaction Report:

(Note: With the exception of the correction of typographical or spelling errors that could be a source of ambiguity, letters and reports are not edited. The original formatting of letters and referee reports may not be reflected in this compilation.)

1st Editorial Decision 01 October 2012

Thank you for the submission of your article "Strategy for creation of clinical grade hESC line banks that HLA-match target population" to EMBO Molecular Medicine. We have now heard back from the referees who agreed to evaluate your manuscript.

You will see that the reviewers are overall positive about your article, but have some concerns that we would like you to convincingly address in a revision of the article.

I look forward to reading your revised manuscript as soon as possible.

Yours sincerely,

Editor
EMBO Molecular Medicine

***** Reviewer's comments *****

Referee #1:

In this manuscript the authors decribe the feasibility of determining the HLA type from embryos donated for stem cell research using the same meythods as are generally used for pre-implantation digagnostics. The message as such is not scientifically new because HLA has been studied frequently from preimplantation embryos with the aim to get matching umbilical cord blood stem cells and at the same time exclude a known hematological disease requiring stem cell transplanatation that has been diagnosed in an older sibling. But this group has also derived clinical

© EMBO

grade human embryonic stem cell (hESC) lines from embryos with pre-determined HLA-type. This enables avoiding the expensive derivation of redundant hESC lines and use the funds for deriving clinical grade HLA-different lines. This is not an unexpected result, but it was worth while to show that it works. The article is comprehensive and well written

Referee #2:

It is no doubt that HLA haplotyping during pre-derivation would be a sensible solution to minimize the cost for hESC bank for clinical use.

Phi 29-based whole genome amplification (if not, author should specify) of a single blastomere from cleavage-stage embryos (day 3 post fertilization), and genetic haplotyping technique for HLA typing and for diagnosis of Huntington's Disease (HD) are well established method. So the value of this report lies on its social and economical aspect.

I believe a couple of modifications may help to improve the quality of the manuscript before publication.

1. Since authors address the cost management of the bank in this report, they shall show the breakdown of the cost listed in Fig. 1a and 1b or at least refer to explanation on the respective cost. If the total cost of 200,000 USD for clinically usable hES cell line consists of that of master bank (120,000 USD) and working bank (80,000 USD), state clearly in Fig.1a and 1b. The breakdown of the cost will be useful information for ones engaged in banking of this kind. It is not necessarily be in detail, but shall show how much testing cost, labor cost or facility construction and running cost were counted to make this figure.
2. Respective cost shown in Fig. 1a and 1b shall be linked each other to give a clear picture of banking procedure and cost.
3. The two photos of day5 blastocyst shown in Fig.1a look identical. The aim of this schema is that two distinct HLA typing approaches (post- vs pre-derivation) give the striking difference in banking cost. So presenting two distinct approaches by two identical photos might not be appropriate in a conceptual schema like Fig.1b. So I am comfortable if two distinct routs are symbolized by two different photos of day 5 blastocyst.
4. In the last sentence in abstract, authors claimed that they would have reduced their cGMP running cost by around 75%, a saving of approximately 700,000 USD. But this is a result of cost comparison of specific eight established hESC cell lines, and this figure cannot be generalized. The content of abstract shall be understood by itself, (not by reading through the figures in main text) and authors shall revise the sentence accordingly.

1st Revision - authors' response 04 October 2012

Response to reviewers

Referee #1:

In this manuscript the authors describe the feasibility of determining the HLA type from embryos donated for stem cell research using the same methods as are generally used for pre-implantation diagnostics. The message as such is not scientifically new because HLA has been studied frequently from pre-implantation embryos with the aim to get matching umbilical cord blood stem cells and at the same time exclude a known hematological disease requiring stem cell transplantation that has been diagnosed in an older sibling. But this group has also derived clinical grade human embryonic stem cell (hESC) lines from embryos with predetermined HLA-type. This enables avoiding the expensive derivation of redundant hESC lines and use the funds for deriving clinical grade HLA-different lines. This is not an unexpected result, but it was worth while to show that it works. The article is comprehensive and well written

We would like to thank Reviewer 1 for such a positive and complimentary response to our manuscript. The HLA typing from pre-implantation embryos is indeed not scientifically new.

However, current approach requires lengthy genotyping, optimization and validation of each family at the single level. We demonstrated here for the first time that whole genome amplification of genomic DNA from a single blastomere is sufficient for rapid, reliable and accurate single nucleotide polymorphism (SNP)-typing for medium resolution typing by PCR amplification with sequence-specific primers (PCR-SSP) as well as the haplotype status for any disease gene being tested.

Referee #2:

It is no doubt that HLA haplotyping during pre-derivation would be a sensible solution to minimize the cost for hESC bank for clinical use.

Phi 29-based whole genome amplification (if not, author should specify) of a single blastomere from cleavage-stage embryos (day 3 post fertilization), and genetic haplotyping technique for HLA typing and for diagnosis of Huntington's Disease (HD) are well established method. So the value of this report lies on its social and economical aspect.

We would like to thank also Reviewer 2 for such a positive and complimentary response to our manuscript. Although Phi-29 based whole genome amplification of a single blastomere and genetic haplotyping for HLA and Huntington's disease diagnosis are all established methods, our report still represents a technological advance (please, see response to Reviewer 1).

I believe a couple of modifications may help to improve the quality of the manuscript before publication.

1. Since authors address the cost management of the bank in this report, they shall show the breakdown of the cost listed in Fig. 1a and 1b or at least refer to explanation on the respective cost. If the total cost of 200,000 USD for clinically usable hES cell line consists of that of master bank (120,000 USD) and working bank (80,000 USD), state clearly in Fig.1a and 1b. The breakdown of the cost will be useful information for ones engaged in banking of this kind. It is not necessarily be in detail, but shall show how much testing cost, labor cost or facility construction and running cost were counted to make this figure.

We thank Reviewer for this suggestion – great idea. We showed breakdown of the costs in a new Table, which is a part of the revised Fig 1. We have also included a breakdown of the tests that are performed in the testing validation stage as we believe this is useful information for those involved in banking of this kind.

2. Respective cost shown in Fig. 1a and 1b shall be linked each other to give a clear picture of banking procedure and cost.

We revised the Figure 1b to make it clearer for the readers.

3. The two photos of day5 blastocyst shown in Fig.1a look identical. The aim of this schema is that two distinct HLA typing approaches (post- vs pre-derivation) give the striking difference in banking cost. So presenting two distinct approaches by two identical photos might not be appropriate in a conceptual schema like Fig.1b. So I am comfortable if two distinct routs are symbolized by two different photos of day 5 blastocyst.

We revised the Figure as suggested using two different Day 5 blastocyst images.

4. In the last sentence in abstract, authors claimed that they would have reduced their cGMP running cost by around 75%, a saving of approximately 700,000 USD. But this is a result of cost comparison of specific eight established hESC cell lines, and this figure cannot be generalized. The content of abstract shall be understood by itself, (not by reading through the figures in main text) and authors shall revise the sentence accordingly.

We agree with the Reviewer and we revised the sentence in abstract as suggested.
