# Supplementary material for: Strategy for the creation of clinical grade hESC line banks that HLA-match a target population
Source: EMBO Mol Med. 2012 Nov 19;5(1):10–7. doi: 10.1002/emmm.201201973 (PMC3569650; doi:10.1002/emmm.201201973)
Supplement: Supplementary file 2 [file emmm0005-0010-SD2.pdf]

Strategy for creation of clinical grade hESC line banks that HLA-match target population

Laureen Jacquet[1†], Emma Stephenson[1†], Robert Collins[2†], Heema Patel[3], Jane Trussler[3], Roaa Al-Bedaery[1], Pamela Renwick[3], Caroline Ogilvie[3], Robert Vaughan[2,4] & Dusko Ilic[1]*

[1]Embryonic Stem Cell Laboratories, Guy's Assisted Conception Unit, Division of Women's Health, King's College School of Medicine, London, UK; [2]Clinical Transplantation Laboratory, GSTS Pathology, Guy's Hospital, London, UK; [3]Guy's & St. Thomas' Centre for Preimplantation Genetic Diagnosis and Genetics Centre, Guy's & St. Thomas' NHS Foundation Trust, London, UK; [4]MRC Centre for Transplantation, King's College London, King's Heath Partners.

[†] These authors contributed equally to the work.

SUPPORTING INFORMATION TABLE OF CONTEST:

Supplementary Figures

Supplementary Figure 1 S2

Supplementary Figure 2 S3

Supplementary Tables

Supplementary Table 1 S4

Supplementary Table 2 S5 - S11

Figure S1. Characterization of our clinical grade hESC lines including fingerprinting, karyotype, pluripotency and differentiation potential. NYD, not yet done.

KCL031

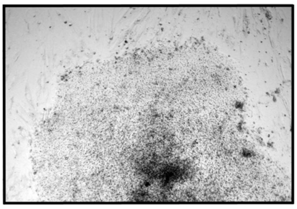

Chr	Marker	Allele1	Allele2
13	D13S252	295	299
	D13S325	293	293
	D13S628	454	457
	D13S634	397	401
18	D18S386	363	382
	D18S390	372	372
	D18S391	209	221
	D18S535	478	486
	D18S819	416	416
	D18S976	476	481
	D18S978	207	211
21	D21S11	245	255
	D21S1409	212	212
	D21S1411	304	320
	D21S1435	184	188
	D21S1446	311	311

Karyotype
46,XY

Pluripotency		
Alkaline phosphatase		nanog
Oct4	TRA-1-60	TRA-1-81

Differentiation in vitro		
ectoderm	mesoderm	endoderm
βIII tubulin	SMA	αFP
PAX6	Brachyury	GATA4

Differentiation in vivo (teratomas)		
ectoderm	mesoderm	endoderm
NYD	NYD	NYD
NYD	NYD	NYD

KCL032

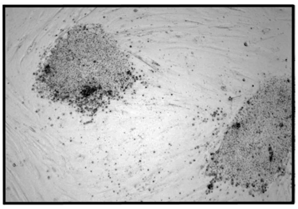

Chr	Marker	Allele1	Allele2
13	D13S252	295	299
	D13S325	281	285
	D13S628	450	457
	D13S634	405	407
18	D18S386	360	386
	D18S390	372	376
	D18S391	217	221
	D18S535	478	482
	D18S819	408	412
	D18S976	476	484
	D18S978	211	211
21	D21S11	241	249
	D21S1409	224	228
	D21S1411	312	316
	D21S1435	184	184
	D21S1446	311	311

Karyotype
46,XX

Pluripotency		
Alkaline phosphatase		nanog
Oct4	TRA-1-60	TRA-1-81

Differentiation in vitro		
ectoderm	mesoderm	endoderm
βIII tubulin	SMA	αFP
PAX6	Brachyury	GATA4

Differentiation in vivo (teratomas)		
ectoderm	mesoderm	endoderm
NYD	NYD	NYD
NYD	NYD	NYD

KCL033

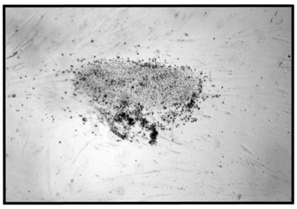

Chr	Marker	Allele1	Allele2
13	D13S252	283	295
	D13S325	285	289
	D13S628	450	457
	D13S634	401	407
18	D18S386	360	386
	D18S390	372	376
	D18S391	217	217
	D18S535	474	478
	D18S819	408	416
	D18S976	472	479
	D18S978	211	211
21	D21S11	241	248
	D21S1409	212	228
	D21S1411	312	316
	D21S1435	184	184
	D21S1446	311	311

Karyotype
46,XX

Pluripotency		
Alkaline phosphatase		nanog
Oct4	TRA-1-60	TRA-1-81

Differentiation in vitro		
ectoderm	mesoderm	endoderm
βIII tubulin	SMA	αFP
PAX6	Brachyury	GATA4

Differentiation in vivo (teratomas)		
ectoderm	mesoderm	endoderm
βIII tubulin	Alcian blue-PAS (cartiliage)	αFP
GFAP	desmin	GATA4

KCL034

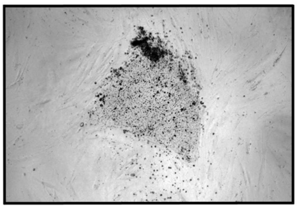

Chr	Marker	Allele1	Allele2
13	D13S252	283	295
	D13S325	285	289
	D13S628	450	458
	D13S634	405	407
18	D18S386	356	386
	D18S390	364	372
	D18S391	217	217
	D18S535	479	482
	D18S819	408	416
	D18S976	473	480
	D18S978	211	215
21	D21S11	245	255
	D21S1409	212	212
	D21S1411	304	320
	D21S1435	184	188
	D21S1446	311	311

Karyotype
46,XY

Pluripotency		
Alkaline phosphatase		nanog
Oct4	TRA-1-60	TRA-1-81

Differentiation in vitro		
ectoderm	mesoderm	endoderm
βIII tubulin	SMA	αFP
PAX6	Brachyury	GATA4

Differentiation in vivo (teratomas)		
ectoderm	mesoderm	endoderm
βIII tubulin	Alcian blue-PAS (cartiliage)	αFP
GFAP	desmin	GATA4

KCL037

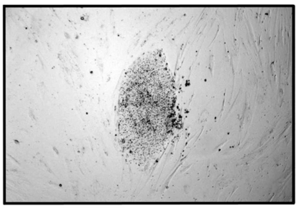

Chr	Marker	Allele1	Allele2
13	D13S252	294	298
	D13S325	285	297
	D13S628	454	461
	D13S634	394	402
18	D18S386	378	389
	D18S390	356	371
	D18S391	209	217
	D18S535	467	479
	D18S819	413	413
	D18S976	478	478
	D18S978	208	212
21	D21S11	242	250
	D21S1409	212	212
	D21S1411	304	338
	D21S1435	186	194
	D21S1446	312	320

Karyotype
46,XY

Pluripotency		
Alkaline phosphatase		nanog
Oct4	TRA-1-60	TRA-1-81

Differentiation in vitro		
ectoderm	mesoderm	endoderm
βIII tubulin	SMA	αFP
PAX6	Brachyury	GATA4

Differentiation in vivo (teratomas)		
ectoderm	mesoderm	endoderm
NYD	NYD	NYD
NYD	NYD	NYD

KCL038

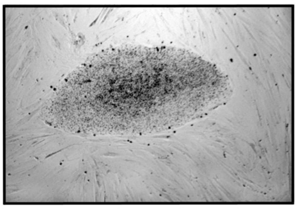

Chr	Marker	Allele1	Allele2
13	D13S252	294	298
	D13S325	297	297
	D13S628	454	461
	D13S634	398	411
18	D18S386	341	378
	D18S390	356	379
	D18S391	217	225
	D18S535	487	491
	D18S819	400	400
	D18S976	471	490
	D18S978	212	216
21	D21S11	256	264
	D21S1409	212	212
	D21S1411	296	317
	D21S1435	186	186
	D21S1446	320	320

Karyotype
46,XY

Pluripotency		
Alkaline phosphatase		nanog
Oct4	TRA-1-60	TRA-1-81

Differentiation in vitro		
ectoderm	mesoderm	endoderm
βIII tubulin	SMA	αFP
PAX6	Brachyury	GATA4

Differentiation in vivo (teratomas)		
ectoderm	mesoderm	endoderm
NYD	NYD	NYD
NYD	NYD	NYD

KCL039

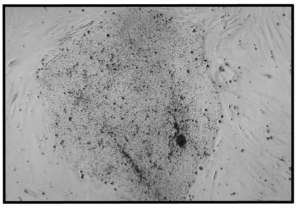

Chr	Marker	Allele1	Allele2
13	D13S252	278	295
	D13S325	285	289
	D13S628	453	457
	D13S634	401	401
18	D18S386	360	379
	D18S390	372	380
	D18S391	213	221
	D18S535	478	482
	D18S819	416	420
	D18S976	467	479
	D18S978	211	215
21	D21S11	240	248
	D21S1409	212	224
	D21S1411	316	320
	D21S1435	180	188
	D21S1446	311	315

Karyotype
46,XY

Pluripotency		
Alkaline phosphatase		nanog
Oct4	TRA-1-60	TRA-1-81

Differentiation in vitro		
ectoderm	mesoderm	endoderm
βIII tubulin	SMA	αFP
PAX6	Brachyury	GATA4

Differentiation in vivo (teratomas)		
ectoderm	mesoderm	endoderm
NYD	NYD	NYD
NYD	NYD	NYD

KCL040

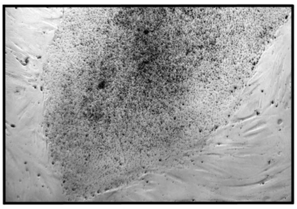

Chr	Marker	Allele1	Allele2
13	D13S252	295	303
	D13S325	285	293
	D13S628	453	457
	D13S634	401	401
18	D18S386	360	382
	D18S390	372	372
	D18S391	217	217
	D18S535	478	494
	D18S819	412	412
	D18S976	476	476
	D18S978	215	219
21	D21S11	248	248
	D21S1409	224	228
	D21S1411	320	328
	D21S1435	188	188
	D21S1446	315	323

Karyotype
46,XX

Pluripotency		
Alkaline phosphatase		nanog
Oct4	TRA-1-60	TRA-1-81

Differentiation in vitro		
ectoderm	mesoderm	endoderm
βIII tubulin	SMA	αFP
PAX6	Brachyury	GATA4

Differentiation in vivo (teratomas)		
ectoderm	mesoderm	endoderm
NYD	NYD	NYD
NYD	NYD	NYD

Figure S2. Characterization of our HD hESC lines including fingerprinting, karyotype, pluripotency and differentiation potential. NYD, not yet done.

KCL012

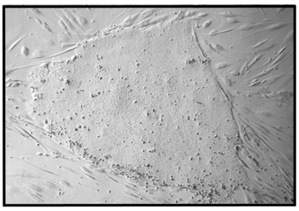

Chr	Marker	Allele1	Allele2
13	D13S252	278	290
	D13S325	292	301
	D13S628	446	457
	D13S634	398	405
18	D18S386	367	383
	D18S390	360	372
	D18S391	217	217
	D18S535	478	478
	D18S819	416	416
	D18S976	480	483
	D18S978	211	219
21	D21S11	244	252
	D21S1409	212	224
	D21S1411	323	323
	D21S1435	184	188
	D21S1446	331	331

Karyotype
46,XY

Pluripotency		
Alkaline phosphatase		nanog
Oct4	TRA-1-60	TRA-1-81

Differentiation in vitro		
ectoderm	mesoderm	endoderm
βIII tubulin	SMA	αFP

Differentiation in vivo (teratomas)		
ectoderm	mesoderm	endoderm
NYD	NYD	NYD
NYD	NYD	NYD

KCL013

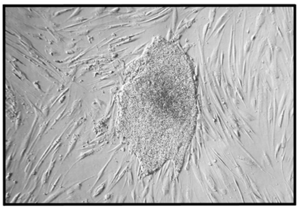

Chr	Marker	Allele1	Allele2
13	D13S252	278	299
	D13S325	297	301
	D13S628	446	457
	D13S634	397	405
18	D18S386	356	356
	D18S390	364	368
	D18S391	217	217
	D18S535	478	499
	D18S819	412	420
	D18S976	480	483
	D18S978	211	211
21	D21S11	241	241
	D21S1409	212	220
	D21S1411	324	324
	D21S1435	184	188
	D21S1446	319	339

Karyotype
46,XY

Pluripotency		
Alkaline phosphatase		nanog
Oct4	TRA-1-60	TRA-1-81

Differentiation in vitro		
ectoderm	mesoderm	endoderm
βIII tubulin	SMA	αFP

Differentiation in vivo (teratomas)		
ectoderm	mesoderm	endoderm
NYD	NYD	NYD
NYD	NYD	NYD

KCL027

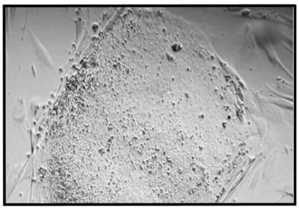

Chr	Marker	Allele1	Allele2
13	D13S252	299	303
	D13S325	289	293
	D13S628	450	457
	D13S634	394	405
18	D18S386	356	386
	D18S390	372	372
	D18S391	217	217
	D18S535	486	486
	D18S819	412	420
	D18S976	473	483
	D18S978	211	211
21	D21S11	245	245
	D21S1409	212	220
	D21S1411	312	324
	D21S1435	193	193
	D21S1446	311	315

Karyotype
46,XY

Pluripotency		
Alkaline phosphatase		nanog
Oct4	TRA-1-60	TRA-1-81

Differentiation in vitro		
ectoderm	mesoderm	endoderm
βIII tubulin	SMA	αFP
PAX6	Brachyury	GATA4

Differentiation in vivo (teratomas)		
ectoderm	mesoderm	endoderm
βIII tubulin	Alcian blue-PAS (cartiliage)	αFP
GFAP	desmin	GATA4

KCL028

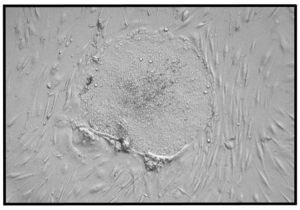

Chr	Marker	Allele1	Allele2
13	D13S252	299	303
	D13S325	289	293
	D13S628	450	458
	D13S634	394	405
18	D18S386	356	386
	D18S390	372	372
	D18S391	217	217
	D18S535	486	486
	D18S819	412	420
	D18S976	473	483
	D18S978	211	211
21	D21S11	244	244
	D21S1409	212	220
	D21S1411	312	324
	D21S1435	192	192
	D21S1446	311	315

Karyotype
46,XY

Pluripotency		
Alkaline phosphatase		nanog
Oct4	TRA-1-60	TRA-1-81

Differentiation in vitro		
ectoderm	mesoderm	endoderm
βIII tubulin	SMA	αFP
PAX6	Brachyury	GATA4

Differentiation in vivo (teratomas)		
ectoderm	mesoderm	endoderm
βIII tubulin	Alcian blue-PAS (cartiliage)	αFP
GFAP	desmin	GATA4

KCL036

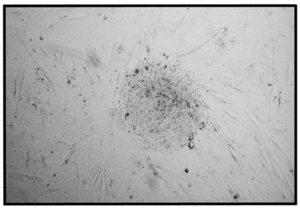

Chr	Marker	Allele1	Allele2
13	D13S252	278	299
	D13S325	293	301
	D13S628	453	453
	D13S634	397	401
18	D18S386	379	382
	D18S390	368	372
	D18S391	209	217
	D18S535	478	482
	D18S819	400	408
	D18S976	476	483
	D18S978	211	211
21	D21S11	249	259
	D21S1409	216	224
	D21S1411	312	316
	D21S1435	188	188
	D21S1446	311	325

Karyotype
46,XX

Pluripotency		
Alkaline phosphatase		nanog
Oct4	TRA-1-60	TRA-1-81

Differentiation in vitro		
ectoderm	mesoderm	endoderm
βIII tubulin	SMA	αFP
PAX6	Brachyury	GATA4

Differentiation in vivo (teratomas)		
ectoderm	mesoderm	endoderm
NYD	NYD	NYD
NYD	NYD	NYD

Supplementary Table 1. HLA haplotypes for our clinical grade lines.
Medium resolution haplotyping was selected as an equivalent to that used for solid organ donation with the addition of immunosuppression.

hESC line	HLA-A*	HLA-B*	HLA-Bw	HLA-C*	HLA-DRB1*	HLA-DRB3/4/5*	HLA-DQB1*
**KCL031**	02	51	4	14:01/07N/11/14	11	3*02	03:01/19/21/22/24
	24	52:01/04/07/13		12:02/17/22/30	15	5*01	06:01
**KCL032**	02	15	6	03	04:07	4*01	03:01/19/21/22/24
	11	51	4	04	13:02/39/73/96	3*03	06:04/27/34/36/38/39
**KCL033**	11	44	4	16	04:07	4*01	03:01/19/21/22/24
	29	51		04	07		02:02
**KCL034**	11	44	4	16	04:07	4*01	03:01/19/21/22/24
	29	51		04	07		02:02
**KCL037**	02	35	6	03	01	3*03	05:01
	03	40		04	13:02/39/73/96		06
**KCL038**	03	07	6	03	14	3*02	05:03
	11	15		07	15	5*01	06:02/11/16/33
**KCL039**	01	35:02/83	4	04	3	3*01/02	02:01/04/05
	24	49:01/06/08		07			
**KCL040**	03	07	6	03	04:04/23/42/56/70	4*01, 5*01	03:02
	24	15		07	15		06:02/11/14/26N

Supplementary Table 2. Self-declared ethnicity of all patients attending Guy's ACU for treatment in 2010.

Each couple completes a standard registration form at the beginning of treatment, in which they declare their ethnicity in an open format. Results are presented exactly as written without amalgamation.

Ethnicities of all PGD patients
(from 165 PGD patients, 158 females and 156 males stated their ethnicity)

Ethnicity	No.	Ethnicity	No.
**FEMALE**		**MALE**	
White British	99	White British	103
White Irish	13	White Irish	12
Indian	7	Indian	5
Chinese	5	Chinese	5
Black African	4	Black African	4
Arab	3	Arab	3
White Welsh	2	Pakistani	3
White	2	White Welsh	2
Japanese	2	White Italian	2
Pakistani	2	White	1
Asian	1	Asian	1
Sri Lankan	1	Sri Lankan	1
Bangladeshi	1	Bangladeshi	1
Black British	1	Black British	1
Chilean	1	Greek Cypriot	1
Greek Cypriot	1	Jewish	1
Iranian	1	White S.african	1
Jewish	1	White Netherlands	1
Latino/S.American	1	White Lithuanian	1
Vietnamese	1	White NewZealand	1
White Finnish	1	White European	1
White French	1	White German	1
White NewZealand	1	White Scottish	1

White Polish	1	White Uruguay	1
White Russian	1	Mixed (Irish & Italian)	1
White Scottish	1	Mixed (White & Black)	1
White Serbian	1		
White Uruguay	1		
Mixed (White & Black)	1		
**Total**	158	**Total**	156

Ethnicities of all ICSI & IVF patients:

Female **Total 873**

**Ethnicity**	**No.**	**Ethnicity**	**No.**
Afghani	6	White Columbian	3
African American	1	Mixed (Black and white)	2
Albanian	1	Moroccan	1
Algerian	1	Other White	1
Anglo-Indian	1	Pakistani	20
Arab	2	Polish/Turkish	1
Asian	31	Portuguese	5
Asian Mauritian	1	White Romanian	6
Asian Filipino	7	White South African	10
Sri Lankan	19	Serbian	1
Indian	94	Somalian	3
Japanese	9	Sudanese	1
Asian/Oriental	1	Tamil	2
Bangladeshi	7	Turkish	1
Black African	93	Vietnamese	3
Black Caribbean	14	West Indian	1
Black British	22	White	1
Black Mixed	1	White/Asian	1
Black Saotome	1	German/Portuguese	1
Black/West Indian	1	White Polish	12
Brazilian	6	White American	8
Bulgarian	6	White Australian	7
Caribbean/Indian	1	White Azerbaijan	1

Chinese	20	White British	297
Croatian	1	White Hungarian	1
European/Asian	1	White Italian	11
White French	11	White European	9
Greek	5	White German	7
Greek/Irish	1	White Irish	23
Hispanic	3	White Latvian	1
White British/Australian	1	White Lithuanian	6
Iranian	4	White Moldovan	2
Iraqi	2	White Netherlands	1
Jewish	1	White New Zealand	4
Korean	1	White Norwegian	2
Kuwaiti	1	White Russian	1
Kyrgyz	1	White Serbian	1
Latin American	3	White Slovakian	2
Chilean	1	White Spanish	7
White Canadian	3	White Ukrainian	4
Malaysian	2	White Uzbekistani	1
Mauritian	2	White/Indian	1
Middle Eastern	2	White Scandinavian	1
Mixed	4	White Czech	1
White Dutch	1	White Estonia	1

Appendix B

Ethnicities of all ICSI & IVF patients:

<u>Male</u> **Total 870**

**Ethnicity**	**No.**	**Ethnicity**	**No.**
Afghani	5	Mixed (Black and white)	1
Anglosaxon	1	Moroccan	1
Albanian	2	Other White	3
Algerian	4	Pakistani	22
Armenian	1	Egyptian	1
Arab	1	Portuguese	7
Asian	27	White Romanian	4
Asian Mauritian	1	White South African	12
Asian Filipino	7	Serbian	1
Sri Lankan	19	Somalian	3
Indian	88	Sudanese	1
Japanese	2	Tamil	2
Asian/Oriental	1	Turkish	2
Bangladeshi	8	Vietnamese	2
Black African	87	West Indian	1
Black Caribbean	15	White	3
Black Jamaican	2	Nepalese	1
Black other	1	White Scottish	2
Black British	16	White/Asian	2
Armenian	1	White Polish	12
Black Saotome	1	White American	8
Brazilian	2	White Australian	4

Bulgarian	6	White Swedish	1
Indian/Portuguese	1	White British	346
Chinese	12	White Italian	12
Croatian	1	White European	11
White French	5	White German	5
Greek	3	White Irish	30
Palestinian	1	White Mediterranean	1
Hispanic	1	White Lithuanian	3
Kazakh	1	White Moldovan	1
Iranian	4	White Netherlands	4
Iraqi	2	White New Zealand	6
Jewish	1	White Russian	2
Latino	1	White Welsh	2
Kuwaiti	1	White Slovakian	3
Latin American	1	White Spanish	1
White Canadian	3	White Ukrainian	3
Malaysian	1	White Estonia	1
Mauritian	1		
Middle Eastern	2		
Mixed	4		
